# Dipeptidyl Peptidase‐4 Inhibitors Attenuate Endothelial Function as Evaluated by Flow‐Mediated Vasodilatation in Type 2 Diabetic Patients

**DOI:** 10.1161/JAHA.112.003277

**Published:** 2013-02-22

**Authors:** Makoto Ayaori, Naotsugu Iwakami, Harumi Uto‐Kondo, Hiroki Sato, Makoto Sasaki, Tomohiro Komatsu, Maki Iizuka, Shunichi Takiguchi, Emi Yakushiji, Kazuhiro Nakaya, Makiko Yogo, Masatsune Ogura, Bonpei Takase, Takehiko Murakami, Katsunori Ikewaki

**Affiliations:** 1 Division of Anti‐aging and Vascular Medicine, Department of Internal Medicine, National Defense Medical College, Tokorozawa, Japan (M.A., H.U.K., M.S., T.K., M.I., S.T., E.Y., K.N., M.Y., M.O., K.I.); 2 Department of Preventive Medicine and Public Health, National Defense Medical College, Tokorozawa, Japan (H.S.); 3 Department of Intensive Care, National Defense Medical College Hospital, Tokorozawa, Japan (B.T.); 4 National Cerebral and Cardiovascular Center, Osaka, Japan (N.I.); 5 Japan Self Defense Force Maizuru Hospital, Maizuru, Japan (N.I., T.M.)

**Keywords:** DPP‐4 inhibitors, endothelial function, flow‐mediated vasodilatation, type 2 diabetes

## Abstract

**Background:**

Endothelial dysfunction is an independent predictor for cardiovascular events in patients with type 2 diabetes (T2DM). Glucagon like peptide‐1 (GLP‐1) reportedly exerts vasodilatory actions, and inhibitors of dipeptidyl peptidase‐4 (DPP‐4), an enzyme‐degrading GLP‐1, are widely used to treat T2DM. We therefore hypothesized that DPP‐4 inhibitors (DPP‐4Is) improve endothelial function in T2DM patients and performed 2 prospective, randomized crossover trials to compare the DPP‐4I sitagliptin and an α‐glucosidase inhibitor, voglibose (in study 1) and the DPP‐4Is sitagliptin and alogliptin (in study 2).

**Methods and Results:**

In study 1, 24 men with T2DM (46±5 years) were randomized to sitagliptin or voglibose for 6 weeks without washout periods. Surprisingly, sitagliptin significantly reduced flow‐mediated vasodilatation (FMD; −51% compared with baseline, *P*<0.05) of the brachial artery despite improved diabetic status. In contrast, voglibose did not affect FMD. To confirm this result and determine whether it is a class effect, we conducted another trial (study 2) to compare sitagliptin and alogliptin in 42 T2DM patients (66±8 years) for 6 weeks with 4‐week washout periods. Both DPP‐4Is improved glycemic control but significantly attenuated FMD (7.2/4.3%, *P*<0.001, before/after sitagliptin; 7.0/4.8%, *P*<0.001, before/after alogliptin, respectively). Interestingly, FMD reduction was less evident in subjects who were on statins or whose LDL cholesterol levels were reduced by them, but this was not correlated with parameters including DPP‐4 activity and GLP‐1 levels or diabetic parameters.

**Conclusions:**

Our 2 independent trials demonstrated that DPP‐4 inhibition attenuated endothelial function as evaluated by FMD in T2DM patients. This unexpected unfavorable effect may be a class effect of DPP‐4Is.

**Clinical Trial Registration:**

URL: http://center.umin.ac.jp, Unique Identifiers: UMIN000005682 (sitagliptin versus voglibose) and UMIN000005681 (sitagliptin versus alogliptin).

## Introduction

Type 2 diabetes (T2DM) is an important risk factor for the development of cardiovascular diseases (CVD).^1^ In recent years, accumulating evidence has demonstrated that endothelial function is impaired,^2–3^ and it is an independent predictor for future cardiovascular events in patients with T2DM.^4^ A number of underlying mechanisms are reportedly involved in endothelial dysfunction in diabetes, in which the bioavailability of endothelium‐derived nitric oxide (NO) is reduced. In endothelial cells, hyperglycemia attenuates NO production by inhibiting endothelial nitric oxide synthase (eNOS) and stimulates superoxide anions, which directly quench NO to form peroxinitrite, resulting in uncoupling of eNOS. Furthermore, compared with controls, patients with T2DM or insulin resistance have been observed to have higher levels of serum asymmetric dimethylarginine,^5–6^ an endogenous NO inhibitor. Overall, endothelial dysfunction in T2DM leads to vasoconstriction, inflammation, and thrombosis, which in turn induce atherogenesis.

Glucagon like peptide‐1 (GLP‐1), in addition to enhancing glucose‐stimulated insulin secretion by pancreatic beta cells, reportedly has beneficial effects on vascular function. Several researchers have reported its direct vasodilatory effect,^7–10^ which was exerted via GLP‐1 receptor (GLP‐1R)–dependent‐^8^ and –independent^10^ pathways. GLP‐1 and its analogues have indeed been observed to improve endothelial function in humans^11–14^ and in animal models.^10,15^ Inhibitors of dipeptidyl peptidase‐4 (DPP‐4), an enzyme degrading active GLP‐1(7‐36) to inactive GLP‐1(9‐36), are widely used to treat T2DM, and recent studies observed that the DPP‐4 inhibitor (DPP‐4I) des‐fluoro‐sitagliptin enhanced acetylcholine‐induced endothelium‐dependent vasodilatation using mice aortic rings,^16^ and vildagliptin, another DPP‐4I, improved endothelial function as shown by measurement of forearm blood flow during acetylcholine (Ach) infusion in T2DM patients.^17^ However, it remains unclear whether this property of vildagliptin is a class effect of DPP‐Is and would be observed when measuring flow‐mediated vasodilatation (FMD) of the brachial artery, which, because of its noninvasive nature, is more widely used for evaluation of endothelial function than forearm blood flow measurement using Ach infusion.

In the present research, to our surprise, DPP‐4Is attenuated endothelial function as evaluated by FMD in T2DM patients in 2 independent prospective, randomized, crossover trials. This unexpected unfavorable effect was independent of serum GLP‐1 level or DPP‐4 activity.

## Methods

### Study 1: Comparison of Sitagliptin and Voglibose—Study Design and Population

This study consisted of a 4‐week prestudy observation period, followed by 2 sets of a 6‐week treatment period. Study subjects were either those who had been treated with oral antidiabetic drugs at Japan Self Defense Force Maizuru Hospital or those who had been referred to the hospital from 7 Medical Service Units in the Maizuru area because of impaired glucometabolic parameters discovered during annual health checkups and eventually diagnosed to have T2DM or borderline T2DM. Inclusion criteria were age ≥20 and <75 years, hemoglobin A1c (HbA1c) >6.2%, fasting blood glucose level >110 mg/dL despite medical therapy including lifestyle or/and pharmacological interventions for ≥8 weeks. Exclusion criteria included (1) type 1 diabetes; (2) secondary diabetes; (3) poorly controlled diabetes (HbA1c ≥10.0%); (4) history of stroke, acute coronary syndrome, or any cardiovascular diseases requiring inpatient treatment within 6 months, end‐stage renal disease, hepatic dysfunction (either level of aspartate aminotransaminase or alanine aminotransferase >3 times normal limits), malignancies, or inflammatory diseases. To investigate the effect of sitagliptin, a DPP‐4I, we compared it with an α‐glucosidase inhibitor, voglibose, which reportedly improves endothelial function.^18–19^ After the observation period, 26 eligible patients (mean age, 46±5 years) were randomized to either sitagliptin (50 mg/day) or voglibose (0.9 mg/day, TID) treatment with crossover to the other drug. Measurement of FMD/nitroglycerin (NTG)–mediated vasodilatation (NMD) and blood/urine sampling were performed at baseline and after 6 weeks on the respective treatments. Thereafter, the treatments were switched, and the same protocol was applied for another 6 weeks.

### Study 2: Comparison of Sitagliptin and Alogliptin—Study Design and Population

This study also consisted of a 4‐week prestudy observation period, followed by 2 sets of a 6‐week treatment period. A washout period (4 weeks) was inserted between the first and second treatment periods. Study subjects were recruited among outpatients of the National Defense Medical College Hospital, among whom some were already being treated for T2DM with oral antidiabetic drugs. Other subjects were those referred from other clinics because they had potential T2DM. Inclusion criteria were age ≥20 and <75 years, HbA1c >6.5%, fasting blood glucose levels >126 mg/dL, or postprandial glucose levels >200 mg/dL despite medical therapy including lifestyle change/medications for ≥8 weeks. Exclusion criteria were the same as those for the study comparing sitagliptin and voglibose. After the observation period, 45 eligible patients (mean age, 66±7 years) were randomized to either sitagliptin (50 mg/day) or alogliptin (25 mg/day) treatment with crossover to the other drug. Measurement of FMD and blood/urine sampling were performed at baseline and after 6 weeks on the treatments. Thereafter, the treatments were ceased for 4 weeks and then switched, and the same protocol was applied for another 6 weeks.

Other medications, including antidiabetic, antihypertensive, or lipid‐lowering drugs, were maintained throughout both studies. They were approved by the ethics committee of the National Defense Medical College, and written informed consent was obtained from each subject.

### Sample Size Computations

For study 1, the sample size was calculated on the basis of a previous study comparing the effect on FMD of voglibose and miglitol, another α‐glucosidase inhibitor,^18^ because of limited information on the effect of DPP‐4Is on FMD. Assuming that DPP‐4Is have a effect similar to miglitol and expecting a 2.3% difference with a standard deviation of 2.0 between sitagliptin and voglibose, a total of 26 subjects would be required at a 5% significance level and 80% power.

We calculated the sample size for study 2 on the basis of the observation that in study 1, prescription of sitagliptin caused a reduction in FMD. On this basis, a total of 40 patients were required to detect a reduction of 2.3% in FMD after treatment, with a standard deviation of 3.6, with 80% power and statistical significance of 5%. Sample‐size calculations were performed using SAS PROC POWER procedure (SAS Institute Inc, Cary, NC).

### Assessment of Endothelial Function

Endothelial function was assessed by FMD of the brachial artery. After measurement of systolic and diastolic blood pressure, FMD was measured noninvasively using a high‐resolution ultrasound apparatus with a 7.5‐MHz linear array transducer (Aplio SSA‐770A, Toshiba Co Ltd) according to the guidelines of the International Brachial Artery Reactivity Task Force.^20^ All measurements were performed in the morning from 9 am to 11 am, before taking the drugs, in a temperature‐controlled room (25°C) with the subject in a fasting, resting, and supine state. Electrocardiograms were monitored continuously. The subject's dominant arm (right) was immobilized comfortably in the extended position to allow consistent access to the brachial artery for imaging. The vasodilatation responses of the brachial artery were observed using a previously validated technique.^21^ For each subject, optimal brachial artery images were obtained between 2 and 10 cm above the antecubital fossa. First, baseline 2‐dimensional (2D) images were obtained, and after measurement of the baseline artery diameter, a narrow‐width blood pressure cuff was inflated on the most proximal part of the forearm to an occlusive pressure (200 mm Hg) for 5 minutes to induce hyperemia. The position of the ultrasound transducer was carefully maintained throughout the procedure. The cuff was then deflated rapidly, and 2D images of the artery were obtained for 30 to 120 seconds after deflation. In the study comparing sitagliptin and voglibose, we measured endothelium‐independent vasodilatation due to administration of NTG (0.3 mg) using the same method. NMD was measured before (baseline) and 240 to 300 seconds after NTG administration. Throughout the study, FMD and NMD were examined by cardiologists (1 in study 1 and 2 in study 2) who were blinded to the treatment regimen of each subject, using the same ultrasound apparatus and probe set for all measurements. Each subject was examined by the same examiner throughout the study. All images were recorded as movie files on a hard‐disk recorder for later analysis. To manually measure vasodilator responses in each patient's artery, movies were played back and a 10‐ to 20‐mm segment was identified for analysis using anatomic landmarks. To select images reproducible for the same point in the cardiac cycle, images at peak systole were identified, and the diameter of the artery was digitized using a caliper function of the ultrasound apparatus. For each condition (baseline, FMD, baseline before NMD, and after NMD), 3 separate images from 3 different cardiac cycles were digitized and their average segment diameters determined. Both FMD and NMD are expressed as percentage change from baseline to peak dilation. The intra‐ and interobserver variability (coefficient of covariance) for repeated diameter measurements at baseline and reactive hyperemia or NMD in the brachial artery were both <3%.^21^

### Blood/Urine Sampling

Venous blood and urine samples for measurement of biochemical parameters were obtained in the morning after an overnight fast before taking the treatment drugs. The subjects were advised to take the DPP‐4Is after breakfast. For measurement of GLP‐1 levels, plasma was obtained in vacutainers containing a DPP‐4I (Millipore, Bedford, MA) according to the manufacturer's instructions. The vacutainers were immediately transferred on ice after sampling, centrifuged, isolated, and stored at −70°C until further analyses.

### Biochemical Analyses

Serum total cholesterol (TC), triglycerides (TG), high‐density lipoprotein (HDL)‐C, glucose, and creatinine levels were determined by standard enzymatic methods. LDL‐C levels were calculated using the Friedewald formula. Hemoglobin A1c (HbA1c) was determined using high‐performance liquid chromatography with calibration using Japan Diabetes Society (JDS) Lot 2.^22^ The equation used for conversion from HbA1c (JDS) to HbA1c (National Glycohemoglobin Standardization Program [NGSP]) values was as follows: NGSP (%)=JDS (%)+0.4%. Serum glycated albumin was determined by enzymatic methods using albumin‐specific protease, ketoamine oxidase, and albumin assay reagents (Lucica GA‐L; Asahi Kasei Pharma, Tokyo, Japan; CV=0.63% to 0.93% intra‐assay, 0.56% to 0.67% interassay).^23^ Plasma insulin levels were measured by chemiluminescent enzyme immunoassay. Serum malondialdehyde‐modified LDL (MDA‐LDL) levels were determined by enzyme‐linked immunosorbent assay (ELISA; CV=6.2% to 9.5% intra‐assay, 2.6% to 11.8% interassay).^24^ Serum high‐sensitivity C‐reactive protein (hsCRP) levels were measured using a BNII nephelometer (Dade Behring, Germany). Serum 8‐hydroxy‐2′‐deoxyguanosine (8‐OHdG) and nitrate/nitrite (NOx) levels were determined by ELISA and a colorimetric assay using commercially available kits—JaICA (Fukuroi, Japan; CV=1.8% to 5.5% intra‐assay, 2.8% to 7.9% interassay) and Calbiochem (La Jolla, CA), respectively—and sera filtered though a Microcon Centrifugal Filter Device YM‐10 (Millipore, Billerica, MA). Serum asymmetric dimethylarginine (ADMA) was determined by ELISA (Immundiagnostik, Bensheim, Germany; CV=6.5% to 7.0% intra‐assay, 6.5% to 7.0% interassay). Serum DPP‐4 activity was measured using a commercially available kit (Enzo Life Sciences, Tokyo, Japan). Urinary albumin was determined by a turbidimetric immunoassay and the urinary creatinine concentration measured by a standard laboratory method. Urinary albumin excretion was estimated by calculating the albumin:creatinine ratio (ACR). To determine the levels of active GLP‐1(9‐37)amide in DPP‐4I‐treated plasma, C18 reverse‐phase extraction columns (MicroSpin C18; GL Science, Tokyo, Japan) were used. The eluent from the extraction columns was evaporated and reconstituted with distilled water, followed by determination of active GLP‐1 using an ELISA kit (ALPCO, Salem, NH; CV=4.7% to 10.7% intra‐assay, 9.6% to 17.6% interassay).

### Statistical Analyses

Data are presented as mean with standard deviation (SD) or median with interquartile range for continuous variables and as frequency with percentage for categorical variables. Baseline characteristics were summarized by treatment sequences and compared using an unpaired *t* test for continuous variables and Fisher's exact test for categorical variables.

Treatment effects on body weight, blood pressure, biochemical parameters, and endothelial functions were assessed using a linear mixed model. Period and sequence were included in the model as fixed effects. Patients within a sequence were included in the model as a random effect. The model was adjusted for sex, age, smoking status, medication with antihypertensive drugs (β‐blockers, calcium channel blockers, angiotensin‐converting enzyme inhibitors, angiotensin receptor blockers, and diuretics), statins, and antidiabetic drugs (biguanides, sulfonylureas, pioglitazone, and α‐glucosidase inhibitors). Hypertension and dyslipidemia were excluded from the model because of interaction between these diseases and drug prescription. Tests for carryover effect and period effect were also performed.

In study 2, a relationship between the change in FMD and each biochemical parameter before and after treatment with DPP‐4Is was assessed using a linear mixed model including patient number as a random effect. The model was adjusted for sex, age, smoking status, and concomitant medications.

For all analyses, a 2‐sided *P<*0.05 was considered statistically significant. All statistical analyses were performed with SAS version 9.3 (SAS Institute Inc, Cary, NC).

## Results

### Study 1: Comparison Between Sitagliptin and Voglibose

#### Baseline characteristics

The baseline characteristics of each group (sitagliptin‐ or voglibose‐first) are shown in Table 1. In each group, there were 3 subjects with borderline T2DM diagnosed according to the Guideline of Japan Diabetes Society. Two subjects in the voglibose‐first group discontinued participation in the study because of gastrointestinal complaints. The remaining subjects were all male patients aged 32 to 59 years. There were no differences between the 2 groups in age, body mass index, HbA1c level, smoking status, medications for diabetes/hypertension, concomitant use of statins/aspirin, or complications, but there was a difference in hypertension, which was significantly more common in the sitagliptin‐first group.

**Table 1.** Baseline Characteristics of Study Subjects—Study Comparing Sitagliptin and Voglibose

	Sitagliptin>Voglibose (n=13)	Voglibose>Sitagliptin (n=11)
Women, n (%)	0 (0)	0 (0)
Age, y	45.9±6.1	45.7±5.8
Body mass index, kg/m^2^	29.4±6.4	27.0±4.0
Hemoglobin A1c, %	6.85±1.12	7.35±2.33
Borderline T2DM	3 (23.1)	3 (27.3)
Current smokers, n (%)	4 (30.8)	7 (63.6)
Hypertension, n (%)	8 (61.5)	2 (18.2)
Dyslipidemia, n (%)	11 (84.6)	5 (45.5)
Coronary artery diseases, n (%)	2 (15.4)	0 (0)
Ischemic stroke, n (%)	0 (0)	0 (0)
Diabetic nephropathy, n (%)	1 (7.7)	1 (9)
Medication for diabetes, n (%)		
Biguanides	3 (23.1)	3 (27.3)
Sulfonylureas	0 (0)	0 (0)
Pioglitazone	1 (7.7)	1 (9.1)
Insulin	0 (0)	0 (0)
Other medication, n (%)		
Antihypertensives	7 (53.9)	2 (18.2)
Statins	4 (30.8)	1 (9.1)
Aspirin	2 (15.4)	0 (0)

T2DM indicates type 2 diabetes mellitus. Diabetic nephropathy was defined by persistent albuminuria with albumin:creatinine ratio >30 mg/g. Values are mean±SD.

#### Biochemical parameters including those of diabetic status and endothelial function

As shown in Table 2, there were no significant differences in parameters between the 2 treatments, except with regard to FMD. Regarding glycemic control, sitagliptin tended to be superior in reducing GA, but not fasting blood glucose or HbA1c. Sitagliptin, but not voglibose, significantly reduced GA and HbA1c levels (*P*=0.001 and 0.0048, respectively) compared with baseline. To our surprise, despite its improvement of glycemic control, FMD was markedly attenuated after sitagliptin treatment (*P*=0.04 versus baseline) compared with voglibose. However, there was no difference in NMD between the treatments, and it was not changed by either of them. Although this study lacked a washout period, carryover effects were not evident (*P* values for all tests for carryover effects >0.1).

**Table 2.** Comparison Between Sitagliptin and Voglibose in Effects on Clinical and Biochemical Parameters and Endothelial Function

	Sitagliptin		Voglibose		*P* Value
	Mean±SD	% Change From Baseline	Mean±SD	%Change From Baseline	
Body weight, kg	86.2±19.1	−5.0	81.6±23.1	1.0	0.228
Systolic blood pressure, mm Hg	131±10	0.9	128±13	−0.9	0.475
Diastolic blood pressure, mm Hg	87±9	2.1	82±11	−3.3	0.093
Biochemical parameters					
Fasting blood glucose, mg/dL	137±43	−5.5	145±43	1.4	0.204
Hemoglobin A1c, %	6.73±1.5	−5.0	6.77±1.38	−4.5	0.845
Glycated albumin, %	16.7±5.5	−7.8	17.4±5.0	−2.8	0.087
Immunoreactive insulin, μU/mL	10.3±9.0	8.4	11.4±12.5	−4.1	0.413
Total cholesterol, mg/dL	200±36	−0.7	213±37	6.8	0.136
LDL cholesterol, mg/dL	123±30	0.4	136±28	10.7	0.193
HDL cholesterol, mg/dL	56.5±13.6	9.2	53.0±10.3	2.3	0.322
Triglycerides, mg/dL	141 (102 to 254)	2.9	136 (104 to 251)	−0.7	0.927
Endothelial function					
Basal diameter before FMD, mm	4.83±0.60	−0.9	4.72±0.57	−3.2	0.272
Peak diameter after FMD, mm	4.94±0.62	−2.9	4.96±0.57	−2.6	0.843
FMD, %	2.13±3.63	−51.1	5.07±3.49	16.4	0.038
Basal diameter before NMD, mm	4.84±0.60	−0.9	4.76±0.57	−2.7	0.394
Peak diameter after NMD, mm	5.59±0.63	−2.0	5.58±0.63	−2.1	0.977
NMD, %	16.2±6.6	−5.8	18.0±6.3	4.8	0.394

SD indicates standard deviation; LDL, low‐density lipoprotein; HDL, high‐density lipoprotein; FMD, flow‐mediated vasodilatation; NMD, nitroglycerin‐mediated vasodilatation. Values are mean±SD except for triglycerides (median [interquartile range]). *P* value of sitagliptin vs alogliptin treatment.

### Study 2: Comparison Between Sitagliptin and Alogliptin

#### Baseline characteristics

To confirm the above observation concerning FMD and assess whether it is a class effect of DPP‐4Is, we conducted another crossover trial to compare 2 DPP‐4Is, sitagliptin and alogliptin, in T2DM patients. Table 3 shows baseline characteristics of study 2—sitagliptin. Compared with in study 1, the study subjects were equally mixed in sex; were elderly, with a mean age of 67 years; and had a statistically greater FMD, of 7.2%, at baseline (*P*<0.001, data not shown). Eight study subjects had experienced coronary artery disease, and 1 study subject had experienced ischemic stroke. There were no significant differences in complications and medications between the sitagliptin‐ and the alogliptin‐first groups (Table 3).

**Table 3.** Baseline Characteristics of Study Subjects—Study Comparing Sitagliptin and Alogliptin

	Sitagliptin>Alogliptin (n=20)	Alogliptin>Sitagliptin (n=22)
Women, n (%)	9 (45.0)	11 (50.0)
Age, y	66.4±7.7	67.4±6.7
Body mass index, kg/m^2^	26.7±3.1	26.7±5.4
Hemoglobin A1c, %	7.01±0.65	7.14±0.69
Current smokers, n (%)	4 (20.0)	4 (18.1)
Hypertension, n (%)	17 (85.0)	19 (86.4)
Dyslipidemia, n (%)	20 (100)	17 (77.3)
Coronary artery diseases, n (%)	3 (15.0)	5 (22.7)
Ischemic stroke, n (%)	1 (5.0)	0 (0)
Diabetic nephropathy, n (%)	9 (45.0)	10 (45.5)
Medication for diabetes, n (%)		
Biguanides	7 (35.0)	6 (27.3)
Sulfonylureas	5 (25.0)	8 (36.4)
Pioglitazone	8 (40.0)	7 (31.8)
α2GIs	1 (5.0)	4 (18.2)
Insulin	0 (0)	0 (0)
Other medication, n (%)		
β‐Blockers	3 (15.0)	7 (31.8)
Calcium blockers	10 (50.0)	12 (54.5)
ARBs	13 (65.0)	9 (40.9)
ACEIs	0 (0)	2 (9.1)
Diuretics	4 (20.0)	1 (4.5)
Statins	14 (70.0)	12 (54.5)
Aspirin	7 (35.0)	10 (45.5)

α2GIs indicates α2 glucosidase inhibitors; ARBs, angiotensin receptor blockers; ACEIs, angiotensin‐converting enzyme inhibitors. Diabetic nephropathy was defined by persistent albuminuria with albumin:creatinine ratio >30 mg/g. Values are mean±SD.

### Biochemical parameters including those of diabetic status and endothelial function

**Figure 1.**
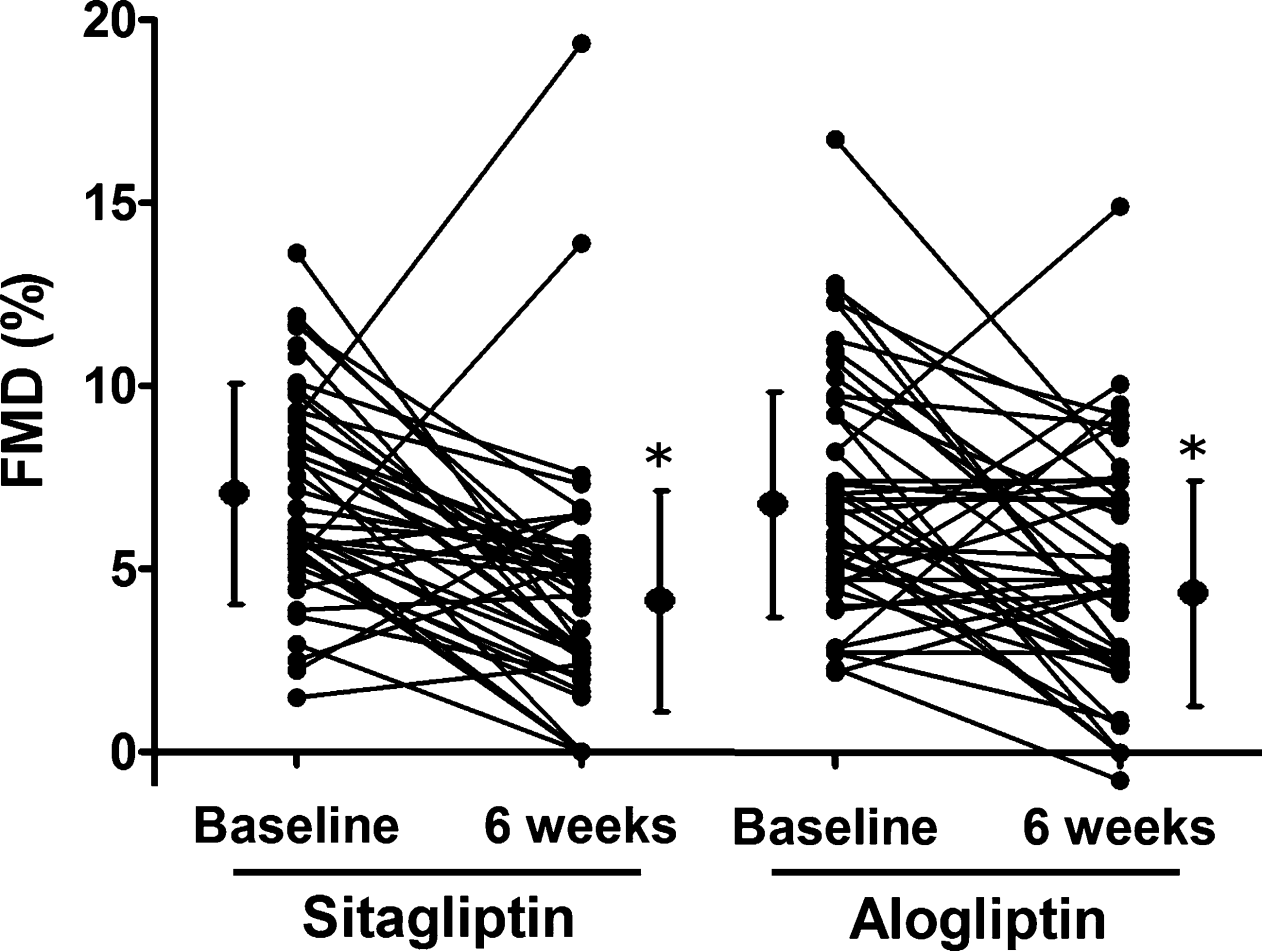
Individual changes in flow‐mediated vasodilatation (FMD) of the brachial artery before and after treatment with sitagliptin and alogliptin. *P<0.001 vs baseline.

Table 4 shows the effects of sitagliptin and alogliptin on various parameters. The primary end points of this study—diabetic status, serum lipids, and FMD—were comparable between the 2 drugs after treatment, except for HDL‐C levels, which were reduced after alogliptin compared with sitagliptin. Alogliptin tended to reduce TC and LDL‐C levels compared with alogliptin. We also analyzed the effects of the DPP‐4Is on other biochemical parameters. Among them, the only difference was seen regarding plasma GLP‐1 levels, with sitagliptin raising levels more than alogliptin. Next, we analyzed changes in various clinical parameters before and after treatment with the DPP‐4Is and found that neither sitagliptin nor alogliptin affected body weight, blood pressure, or heart rate. Both drugs significantly reduced HbA1c, GA, and fasting glucose levels. Despite improving glycemic control, both sitagliptin and alogliptin attenuated FMD, by 39.6% and 31.7%, respectively (Figure 1). As expected, serum DPP‐4 activity was markedly reduced by both sitagliptin and alogliptin. Although neither treatment affected serum insulin levels, sitagliptin, but not alogliptin, significantly increased plasma GLP‐1 levels. Most other parameters—including blood pressure, body weight, serum lipids, ACR, MDA‐LDL, 8‐OHdG, hsCRP, and NOx—were unchanged, although alogliptin significantly reduced TC, LDL‐C, and HDL‐C levels. Levels of ADMA, an endogenous inhibitor of NOS, were significantly increased by sitagliptin, but not by alogliptin. For each clinical/biological parameter, we have included interaction terms for sex and treatment in the model as independent variables. *P* values for interaction terms were not significant in any model.

**Table 4.** Comparisons Between Sitagliptin and Alogliptin in Effects on Clinical/Biochemical Parameters and Endothelial Function

	Sitagliptin		Alogliptin		*P* Value
	Mean±SD	% Change From Baseline	Mean±SD	% Change From Baseline	
Diabetic parameters					
Hemoglobin A1c, %	6.55±0.44	−4.6	6.69±0.56	−3.1	0.126
Glycated albumin, %	16.3±1.4	−9.4	16.9±2.4	−6.7	0.110
Fasting blood glucose, mg/dL	116±16	−8.5	118±19	−10.3	0.524
Serum lipids					
Total cholesterol, mg/dL	193±36	0.3	190±31	−3.4	0.060
LDL cholesterol, mg/dL	113±33	−0.6	111±31	−5.2	0.080
HDL cholesterol, mg/dL	55.9±13.2	1.8	53.3±11.6	−3.8	0.027
Triglycerides, mg/dL	111 (98 to 136)	−10.5	132 (100 to 151)	10.9	0.064
Endothelial function					
Basal diameter before FMD, mm	4.05±0.50	0.8	4.05±0.49	1.4	0.701
Peak diameter after FMD, mm	4.23±0.52	−1.9	4.24±0.51	−0.6	0.266
FMD, %	4.32±3.53	−39.6	4.77±3.27	−31.7	0.459
Body weight, kg	68.0±13.6	−0.6	67.8±11.8	0.1	0.956
Systolic blood pressure, mm Hg	123±13	−1.9	124±14	−0.7	0.854
Diastolic blood pressure, mm Hg	68±10	−5.1	67±9	0.2	0.653
Heart rate, /min	71±14	0	72±10	−1.4	0.745
Other biochemical parameters					
Immunoreactive insulin, μU/mL	9.4±6.0	−9.3	8.9±5.1	−12.6	0.420
DPP‐4 activity, pmol/mL per minute	166±55	−38.6	154±74	−43.8	0.121
GLP‐1, pmol/L	4.14±7.91	139.8	2.38±2.39	14.3	0.045
MDA‐LDL, U/L	127±50	2.0	127±51	0.2	0.975
hsCRP, mg/L	0.64 (0.42 to 1.83)	20.8	0.71 (0.36 to 1.28)	44.9	0.971
8‐OHdG, ng/mL	0.71±0.28	1.3	0.72±0.25	−1.5	0.963
ACR, mg/g Cr	16.8 (8 to 66.3)	−2.9	10.9 (8.0 to 89.7)	−9.2	0.243
Nitrate/nitrite, mol/L	9.40±3.02	3.9	8.99±2.56	−1.0	0.546
ADMA, mol/L	0.48±0.29	8.9	0.44±0.27	−2.9	0.157

SD indicates, standard deviation; LDL, low‐density lipoprotein; HDL, high‐density lipoprotein; FMD, flow‐mediated vasodilatation; DPP‐4, dipeptidyl peptidase‐4; GLP‐1, glucagon‐like peptide‐1; MDA‐LDL, malondialdehyde‐modified LDL; hsCRP, high‐sensitive C‐reactive protein; 8‐OHdG, 8‐hydroxydeoxyguanosine; ACR, urinary albumin:creatinine ratio; ADMA, asymmetric dimethylarginine. Values are mean±SD except for triglycerides, hsCRP, and ACR (median [interquartile range]).

## FMD Reduction Less Remarkable in Subjects Taking Statins and in Those With Reduced LDL‐C Levels After Statin Treatment

Although this was not a predetermined primary question in the study design, we performed an exploratory data analysis to further assess which factors contributed to the attenuation of FMD by the DPP‐4Is. After adjustment for sex, age, smoking status, and concomitant medications, a linear mixed‐model analysis revealed a significant negative correlation between changes in FMD and changes in LDL‐C levels, but not with levels of diabetic parameters, DPP‐4 activity, GLP‐1, serum lipids besides LDL‐C, MDA‐LDL, hsCRP, 8‐OHdG, NOx, or ADMA (Table 5). As shown in Table 6, concomitant statin treatment attenuated DPP‐4I‐induced FMD reduction. Such an association was also observed when other parameters (Table 5) besides LDL‐C were analyzed (data not shown).

**Table 5.** Relationship Between FMD Change and Biochemical Parameters Before and After Treatments With DPP‐4 Inhibitors

	Fixed Effects	*P* Value
Hemoglobin A1c, %	0.67±1.42	0.638
Glycated albumin, %	0.39±0.37	0.303
Fasting blood glucose, mg/dL	0.02±0.02	0.457
DPP‐4 activity, pmol/mL per minute	0.00±0.01	0.950
GLP‐1, pmol/L	−0.13±0.08	0.113
Total cholesterol, mg/dL	−0.04±0.03	0.092
LDL cholesterol, mg/dL	−0.07±0.03	0.021
HDL cholesterol, mg/dL	−0.02±0.07	0.737
Triglycerides, mg/dL	0.00±0.01	0.759
MDA‐LDL, U/L	0.01±0.01	0.159
hsCRP, mg/L	0.34±0.49	0.484
8‐OHdG, ng/mL	−1.02±2.57	0.694
ACR, mg/g Cr	0.00±0.00	0.779
Nitrate/nitrite, μmom/L	−0.08±0.14	0.592
ADMA, μmom/L	−4.38±4.34	0.317

FMD indicates flow‐mediated vasodilatation; DPP‐4, dipeptidyl peptidase‐4; GLP‐1, glucagon‐like peptide‐1; LDL, low‐density lipoprotein; HDL, high‐density lipoprotein; MDA‐LDL, malondialdehyde‐modified LDL; hsCRP, high‐sensitive C‐reactive protein; 8‐OHdG, 8‐hydroxydeoxyguanosine; ACR, urinary albumin:creatinine ratio; ADMA, asymmetric dimethylarginine. For each parameter, a linear mixed‐model analysis was performed, adjusted for sex, age, current smoking, concomitant medications (hypertension, dyslipidemia, and diabetes). Fixed effects are presented as parameter estimates with standard errors.

**Table 6.** Relationship Between Changes in FMD and Those in LDL Cholesterol Levels

	Fixed Effects	*P* Value
LDL cholesterol, mg/dL	−0.07±0.03	0.021
Sex (female)	0.53±0.93	0.575
Age, y	−0.13±0.07	0.052
Current smoking	1.21±1.20	0.317
Concomitant statin treatment	2.64±0.95	0.007
Medication for hypertension	0.48±1.19	0.685
Medication for diabetes (excluding DPP‐4Is)	−0.13±0.87	0.884

FMD indicates flow‐mediated vasodilatation; LDL, low‐density lipoprotein; DPP‐4, dipeptidyl peptidase‐4. Fixed effects are presented as parameter estimates with standard errors.

## Discussion

The present study demonstrated for the first time that DPP‐4Is significantly reduce FMD and indicated that this unfavorable result might be a class effect. The consistency of the results between 2 independent studies with subjects differing in sex and age characteristics supports the validity of this conclusion. Also, the improvement in diabetic status in our 2 studies was similar to that in previous studies, making it also unlikely that the deterioration in FMD was a result of lack of pharmacological action.

This finding is unexpected because GLP‐1^11–13^ and a GLP‐1 analogue^14^ improved endothelial function in previous studies. In 2 of these studies,^11,13^ FMD was investigated in the brachial artery, so a difference in method of evaluating endothelial function could not account for the discrepancy between increasing GLP‐1 levels through infusion of GLP‐1,^11,13^ which enhanced FMD, and increasing active GLP‐1 levels by means of DPP‐4Is (Table 4), which reduced FMD. However, it could be explained by the observations in 2 previous studies that not only metabolically active GLP‐1(7‐36), but also inactive GLP‐1(9‐36)^10^ and the GLP‐1R antagonist exendin(9‐39)^9^ can exert a vasodilatory effect. It was also observed that both GLP‐1(7‐36) and GLP‐1(9‐36) resulted in relaxation responses of the mesenteric artery in mice lacking GLP‐1R,^10^ indicating that inactive GLP‐1(9‐36), and GLP‐1(7‐36) also promote arterial relaxation via GLP‐1R‐independent pathways. Theoretically, DPP‐4 inhibition causes a reduction in serum GLP‐1(9‐36) levels, which is not directly measureable at present. If GLP‐1(9‐36)‐mediated pathways are dominant compared with those stimulated by GLP‐1(7‐36) in vasodilatory action, this could conceivably explain the unfavorable effects of DPP‐4 on endothelial function in the present research.

A recent study by van Poppel et al demonstrated that vildagliptin, another DPP‐4I, improved endothelial function by measuring forearm blood flow during Ach infusion in T2DM patients. Because we did not use vildagliptin in our study, the reason for this discrepancy between their findings and ours is unclear. However, it might be attributable to a difference in methodology. In this regard, Zeiher et al^25^ reported that 3 methods of evaluating endothelial function—FMD, cold pressor test, and Ach‐induced vasodilatation—produced different results in patients with different stages of coronary atherosclerosis, implying that unknown distinct mechanisms could be causing the discrepancy between FMD and Ach‐induced vasodilatation. It might also be a result of a difference in artery type: resistance artery for Ach‐induced vasodilatation and conduit artery for FMD. Furthermore, in our studies FMD was measured before and after the treatments, whereas in van Poppel et al's research it was only measured after the treatments. Thus, their data simply demonstrate the difference in vasodilatation response between vildagliptin and acarbose, which may not be equivalent to demonstrating the effect of vildagliptin on endothelial function.

Based on a literature search,^26^ the present study is the first to find that alogliptin lowered both LDL‐C and HDL‐C levels compared with baseline values (Table 5). From the viewpoint of lipid metabolism, it is unlikely that improved diabetic status caused the reduction in HDL‐C. Regarding the effects of alogliptin on LDL‐C levels, previous studies in Japanese patients with T2DM yielded mixed results; levels were reduced in some^27–28^ but were unchanged in others.^29–30^ We could not find any mechanistic evidence linking DPP‐4I and LDL/HDL‐C, and supposing that such a relationship did exist, we would still not know why alogliptin had this selective effect (Table 5). Furthermore, analysis using a linear mixed model revealed that FMD reduction was less remarkable in subjects taking statins and those with reduced LDL‐C levels after statin treatment (Table 6), and these observations may point to an underlying mechanism because statin treatment and lower levels of LDL‐C are reportedly associated with higher endothelial function.^31–32^ However, this still does not explain the selective effect of alogliptin.

We also demonstrated that the changes in FMD were not associated with parameters involved in FMD (age, sex, smoking, concomitant use of antihypertensives/‐diabetics, and diabetic/lipids parameters) or altered after the DPP‐4I treatments (DPP‐4 activity, GLP‐1 levels, and diabetic parameters). Therefore, overall, future studies are needed to elucidate why LDL‐C/statins should be associated with DPP‐4I‐mediated FMD reduction and determine the related factors.

DPP‐4 reportedly cleaves not only GLP‐1 but also other peptides, such as SDF‐1a, neuropeptide Y, peptide YY, substance P, GLP‐2, and neuropeptide pituitary adenylate cyclase‐activating polypeptide 38, all of which are known to have vascular effects.^33–36^ Although there was no significant correlation between the changes in FMD and those in DPP‐4 activity (Table 6), it is conceivable that known or unknown substrates of DPP‐4 might be involved in DPP4‐I‐induced attenuation of FMD in T2DM patients.

Limitations of this study are worth commenting on. It was not blinded, creating potential bias. To deal with this, measurements were performed by investigators unaware of the randomization status. In addition, all measurements were recorded, and subsequently vessel diameter was analyzed by 2 investigators not knowing the sequence of interventions or assignment to treatments. For study 2, we determined the sample size that would achieve a difference between before treatment and after treatment through sample size calculation based on the results of study 1 and recruited the required number of subjects. Next, both studies were comparisons of 2 active therapies without placebos. Inclusion of placebos would strengthen our arguments. Finally, regarding the associations observed between changes in FMD and those in LDL‐C/statin use, caution should be exercised in interpreting the results because the analysis in this regard was limited by approach, multiple testing, and not being a preset study question.

In conclusion, the present studies independently demonstrated that DPP‐4 inhibition attenuated endothelial function as evaluated by FMD in T2DM patients. Although a recent meta‐analysis found no increase in cardiovascular outcome with another DPP‐4I (saxagliptin),^37^ we should keep this effect in mind, especially in patients with high CVD risk.
